# DNA Methylation-Dependent Dysregulation of GABAergic Interneuron Functionality in Neuropsychiatric Diseases

**DOI:** 10.3389/fnins.2020.586133

**Published:** 2020-09-16

**Authors:** Jenice Linde, Geraldine Zimmer-Bensch

**Affiliations:** ^1^Division of Functional Epigenetics in the Animal Model, Institute for Biology II, RWTH Aachen University, Aachen, Germany; ^2^Research Training Group 2416 MultiSenses – MultiScales, RWTH Aachen University, Aachen, Germany

**Keywords:** inhibitory interneurons, GABA, schizophrenia, autism, cerebral cortex, DNA methylation

## Abstract

Neuropsychiatric diseases, such as mood disorders, schizophrenia, and autism, represent multifactorial disorders, differing in causes, disease onset, severity, and symptoms. A common feature of numerous neuropsychiatric conditions are defects in the cortical inhibitory GABAergic system. The balance of excitation and inhibition is fundamental for proper and efficient information processing in the cerebral cortex. Thus, altered inhibition is suggested to account for pathological symptoms like cognitive impairments and dysfunctional multisensory integration. While it became apparent that most of these diseases have a clear genetic component, environmental influences emerged as an impact of disease manifestation, onset, and severity. Epigenetic mechanisms of transcriptional control, such as DNA methylation, are known to be responsive to external stimuli, and are suspected to be implicated in the functional impairments of GABAergic interneurons, and hence, the pathophysiology of neuropsychiatric diseases. Here, we provide an overview about the multifaceted functional implications of DNA methylation and DNA methyltransferases in cortical interneuron development and function in health and disease. Apart from the regulation of gamma-aminobutyric acid-related genes and genes relevant for interneuron development, we discuss the role of DNA methylation-dependent regulation of synaptic transmission by the modulation of endocytosis-related genes as potential pathophysiological mechanisms underlying neuropsychiatric conditions. Deciphering the hierarchy and mechanisms of changes in epigenetic signatures is crucial to develop effective strategies for treatment and prevention.

## Graphical Abstract

Main conclusions of the minireview.

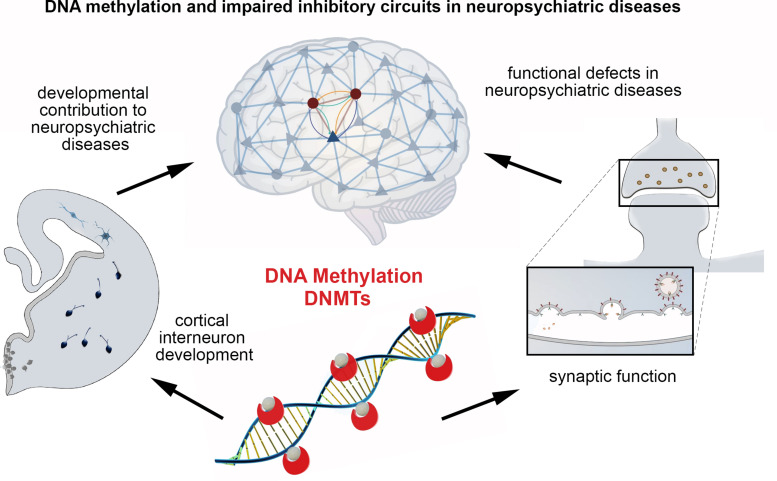

## Introduction

Neuropsychiatric and neurological diseases, like schizophrenia, autism spectrum disorder (ASD), and epilepsy, represent multifactorial disorders, which seem to emerge from a combination of both, genetic predisposition and environmental impacts. For these disorders, evidence was provided that insults during development can likewise contribute to disease manifestation (Tsuang et al., 2001; Brown, 2011; Risch et al., 2014; Bölte et al., 2019). Considering the observation that numerous disease susceptibility genes are linked to neurodevelopmental processes has caused a debate whether schizophrenia, ASD, and epilepsy should be classified as neuropsychiatric or neurodevelopmental diseases (Bolton et al., 1998; Price et al., 2000; Lewis and Levitt, 2002; DiCicco-Bloom et al., 2006; Tuchman and Cuccaro, 2011). Indeed, the age at disease onset can vary tremendously, ranging from patients being few months to several years old. Besides genetic causes (Krebs et al., 2000; Klei et al., 2012; Pourcain et al., 2018), environmental contributions might provide an explanation for the differing onsets as well as for the various degrees of disease severity.

Epigenetic mechanisms of transcriptional control, such as histone modifications, DNA methylation, and non-coding RNAs, provide an attractive hypothesis for the causal relationship of extrinsic incidents and the afflicted persons’ disease development. DNA methylation was reported to be affected distinctively in different neuronal subsets across various neuropsychiatric conditions (Feng and Fan, 2009; Gräff et al., 2011), and emerges to mediate a broad spectrum of biological effects. For this, we focus on DNA methylation here. DNA methylation was traditionally considered a repressive epigenetic mark, impeding transcription factor binding either directly, or indirectly by the action of methyl-CpG-binding domain proteins (Curradi et al., 2002). This view has been challenged by recent studies, suggesting that DNA methylation could also create new motifs for transcription factor binding sites in addition to interaction sites for histone modifying complexes (Smith and Meissner, 2013; Jang et al., 2017). Moreover, DNA methylation is relevant for alternative promoter choice and alternative splicing, giving rise to different protein isoforms (Maunakea et al., 2010, 2013). As DNA methylation was reported to be changed in response to altered neuronal activity (Guo et al., 2011), it could likely mediate or contribute to the integration of external cues into pathological cell features, resulting in impaired neuronal functionality. Moreover, DNA methylation is known to act on diverse aspects of neuronal development, such as differentiation, migration and survival regulation (Hutnick et al., 2009; Chestnut et al., 2011; Pensold et al., 2017; Symmank and Zimmer, 2017), supporting the assumption that it might further be implicated in or mediate the developmental contribution to disease manifestation.

DNA methylation is dynamically regulated by DNA methyltransferases (DNMTs) and ten-eleven translocation (TET) proteins. While DNMTs catalyze cytosine methylation, TET proteins initiate active demethylation by oxidizing 5mC–5hmC and further oxidation forms, which are then actively demethylated by thymine DNA glycosylase (TDG)-mediated base excision repair (Ito et al., 2011; Kaas et al., 2013; Kohli and Zhang, 2013; Wu and Zhang, 2014).

DNMT1 and DNMT3a are the two main DNMTs being expressed in developing and adult neurons. Apart from cell fate specification, neuronal migration, and survival regulation during brain development, DNA methylation and DNMTs were reported to regulate synaptic function and plasticity, being involved in learning and memory regulation (Levenson et al., 2006; Nelson et al., 2008; Meadows et al., 2015, 2016; Sweatt, 2016, 2017). Thereby they seem to exert in part redundant (Feng et al., 2010) but also distinctive functions (Morris et al., 2016). Similarly, TET enzymes were identified to regulate neuronal development (Santiago et al., 2014), synaptic plasticity and memory extinction (Rudenko et al., 2013), as well as gene expression in response to global synaptic activity changes (Yu et al., 2015).

Dysregulated expression of DNMTs and TETs, as well as alterations in both, DNA methylation and demethylation networks, were found in brains of patients suffering from different neuropsychiatric diseases including schizophrenia and ASD, but also epilepsy (Huang and Akbarian, 2007; Zhubi et al., 2009; Guidotti et al., 2011; Dong et al., 2012; Grayson and Guidotti, 2013; Guidotti and Grayson, 2014; Benes, 2015). Based on their implications in healthy brain development and function, it is conceivable that disease-related changes in DNA methylation and demethylation, as well as DNMT and TET function contribute to the symptoms seen in these diseases.

Such symptoms include substantial restrictions in learning and memory formation, disorientation, hallucinations, and problems with communication, which point toward mayor impairments regarding the patients’ sensory cortical information processing. Indeed, one crucial hallmark of neuropsychiatric diseases is defective multisensory integration, which is important for perception, cognitive processing, and complex behaviors, and might thus be the basis for the former mentioned symptoms.

Multisensory processing is known to critically rely on the proper function of inhibitory gamma-aminobutyric acid (GABA) – containing interneurons in the cerebral cortex (Olcese et al., 2013), which synchronize surrounding glutamatergic pyramidal neurons, enabling local neural assemblies (Hensch, 2005; Klausberger and Somogyi, 2008).

The group of cortical inhibitory GABAergic interneurons is enormously diverse in matters of morphological, molecular and functional features (Tremblay et al., 2016). The most abundant subset of cortical interneurons are parvalbumin (PV)-expressing interneurons, which include the fast-spiking chandelier and basket cells (Rudy et al., 2011). Due to the essential role inhibitory interneurons have in cortical information processing (Defelipe et al., 2013), deficits in the development and/or function of GABAergic interneurons are likely implicated in causing or mediating the pathological symptoms in neuropsychiatric diseases. In support of that, alterations in the cortical GABAergic system represent a common denominator of different neuropsychiatric disorders, and human and mouse genetic studies provided evidence for the critical role of altered GABAergic circuit formation in schizophrenia, epilepsy, and autism (Marín, 2012). Especially for schizophrenia and ASD, disturbed interneuron development would explain a variety of the associated pathological symptoms (Lewis and Levitt, 2002; Marín, 2012).

Of note, alterations in DNA methylation signatures of GABA-related genes and/or changed expression of DNMTs in GABAergic interneurons were reported for diverse neuropsychiatric diseases (Veldic et al., 2004; Guidotti et al., 2011). Given that DNMTs and DNA methylation not only regulate cortical interneuron functionality (Pensold et al., 2020) but also their development (Pensold et al., 2017; Symmank et al., 2018, 2020), an implication of altered DNA methylation in inhibitory interneurons for disease manifestation seems plausible. On the other hand, a dysregulated epigenetic machinery could also constitute a secondary effect of disease progression. To explore the potential of epigenetic key regulators as targets for therapeutic interventions, we need to draw a conclusive picture, shedding light on the detailed mechanisms underlying transcriptional dysregulation and impaired physiology of the different neuronal subtypes in neuropsychiatric disorders.

## Defects of the Gabaergic System Contribute to Neuropsychiatric Disorders

In this paragraph, the evidence for the implication of GABAergic defects in different neuropsychiatric and neurological diseases will be discussed in more detail.

Altered levels of GABA and GABA-mediated inhibition were found *in vivo* using magnetic resonance spectroscopy (MRS) in patients suffering from epilepsy or depression (Chang et al., 2003), and in brain tissue of epileptic patients (Treiman, 2001). Moreover, changes in GABAergic function became apparent in genetic and acquired animal models for epilepsy (Treiman, 2001). Concordantly, both GABA antagonists and therapeutics, that downregulate GABA synthesis, can elicit seizures in otherwise unaffected patients. On the other hand, GABA agonists and GABA-mimetic agents act as anticonvulsants (Treiman, 2001) and antidepressants (Sanacora et al., 2000), underlining the implication of GABA in eliciting neurological symptoms.

Altered GABA levels were further detected in post mortem brains of schizophrenia patients (Benes, 2015). In line with this, people suffering from schizophrenia were found to have reduced inhibitory interneuron numbers (Bakhshi and Chance, 2015). On a molecular level, the expression levels of GAD67 (an isoform of the GABA-producing glutamate decarboxylase) and GAT1 (a GABA membrane transporter) mRNAs were found to be noticeably decreased (Lewis et al., 2005). Accordingly, inhibitory cortical interneurons displayed lower neurotransmitter levels and reduced firing capacities in these patients. The following reduction of inhibition of excitatory neurons leads to a systemic disinhibition, resulting in abnormally high activity levels (Bakhshi and Chance, 2015). These aberrant properties were particularly striking in the dorsolateral prefrontal cortex (DLPFC). Additionally, a reduction of REELIN on mRNA and protein levels was evident (Impagnatiello et al., 1998). Under physiological conditions, REELIN mediates neuronal migration and positioning (Franco et al., 2011; Sekine et al., 2014). A mis-positioning of neurons in DLPFC might contribute to personality changes, e.g., expressed as extremely disorganized behavior and paranoid thinking, and impairments on tasks of executive function seen in schizophrenic patients (Bakhshi and Chance, 2015). Furthermore, working memory and attention deficits as well reflect dysfunctional DLPFC activity (Lewis et al., 2004, 2005).

Research on the molecular basis of ASD embraces both investigating ASD and underlying causes in general as well as the role of individual genes, whose aberrant expression or function lead to ASD as a comorbidity. In patients suffering from ASD, several genetic variations were identified as potential causative factors (Marín, 2012). Albeit some of the affected genes are expressed ubiquitously in the brain, restricting the expression of mutated genes to the GABAergic system is already sufficient to induce the expected phenotype, involving stereotypic and compulsive behavior, motor dysfunction and changes in social behavior (Chao et al., 2010). Also, in different mouse models of ASD, a reduced number of inhibitory interneurons throughout the neocortex was found as a common denominator (Gogolla et al., 2009). Based on this it is not surprising that ASD patients have a high comorbidity with epilepsy (Canitano, 2007). Altered levels of GABA and GABA-related synaptic transmission in ASD patients are probably due to the reported changes in mRNA and protein levels of different GABA-receptor-subunits (Fatemi et al., 2010), and a decrease of available GABA synthesizing enzymes GAD65 and GAD67 (Coghlan et al., 2012).

Overall, the described changes of the GABAergic system in the pathological context render neuropsychiatric diseases “interneuropathies”. In addition to working memory, the GABAergic system decisively influences multisensory integration (Olcese et al., 2013), which is virtually inoperable in patients with neuropsychiatric diseases. Multisensory processing is a key feature of higher cognitive power, enabling an individuum to form coherent multimodal objects out of spatio-temporal cohesive inputs. The ability of primary and higher cortical areas to integrate unisensory information and build a meaningful representation of events is the basis of many cognitive capacities such as learning and the expression of behavior (Murray et al., 2016). Hence, impairments in interneuron development and function as seen in neuropsychiatric diseases likely affect multisensory processing. Thus, the described aberrations in the GABAergic system in patients suffering from neuropsychiatric conditions provide a potential explanation for many of the psychiatric symptoms.

How are these aberrations in the GABAergic system caused or mediated? There is increasing evidence that epigenetic mechanisms of gene regulation like DNA methylation in inhibitory GABAergic interneurons are implicated in the pathophysiology of neuropsychiatric diseases like schizophrenia and autism (Huang and Akbarian, 2007; Connor and Akbarian, 2008). To understand the relevance DNMTs and DNA methylation have in the etiology of neuropsychiatric diseases, their implication in interneuron development and adult function in the healthy brain has to be dissected.

## DNMT Function in Interneuron Development

DNA methyltransferase-dependent DNA methylation in developing interneurons seems to be implicated in schizophrenia, as prenatal stress in mice elevates *Dnmt1* and *Dnmt3a* expression in GABAergic interneurons and induces behaviors indicative of a schizophrenia-like phenotype in their offspring (Matrisciano et al., 2013). Neuronal development is a multifaceted process involving differentiation from neural progenitor cells, migration of post-mitotic neurons to their target regions, morphological maturation, and synapse formation. While in the dorsal telencephalon DNA methylation has been proposed to control the neurogenic versus glial cell fate in cortical progenitors (Miller and Gauthier, 2007), such investigations have not been executed so far for the basal telencephalon, from where inhibitory interneurons originate.

At post-mitotic level, DNMT1 was described to regulate cortical interneuron migration by promoting the migratory morphology and survival (Pensold et al., 2017) in part through modulating *Pak6* and *Lhx1* expression as downstream targets (Symmank et al., 2018, 2019, 2020). Interestingly, these genes are DNA methylation-independently regulated by DNMT1 via a crosstalk with histone modifications (Symmank et al., 2019, 2020). In line with this, DNMTs and DNA methylation were further shown to be essential for the maturation of diverse other neuronal subtypes (Fan et al., 2001; Hutnick et al., 2009; Chestnut et al., 2011; Rhee et al., 2012), including cortical excitatory neurons (Hutnick et al., 2009; Feng et al., 2010), and dentate gyrus neurons (Noguchi et al., 2016).

## DNA Methylation and DNMTs in Interneuron Functionality in Health and Disease

Alterations in DNA methylation signatures of synapse- and GABA-related genes were reported for ASD (Nardone et al., 2017) and schizophrenia (Costa et al., 2003; Veldic et al., 2004; Ruzicka et al., 2007). The pathophysiology of the Rett’s syndrome, a form of ASD with symptoms like impaired language skills, cognitive deficits, and stereotypic behavior (Chahrour and Zoghbi, 2007), can be traced back to a loss-of-function mutation of methyl-CpG-binding protein 2 (MECP2; Amir et al., 1999). Consequently, a systemic MECP2 knockout reproduces many of the neurological symptoms of Rett’s syndrome in mice (Chen et al., 2001; Guy et al., 2001). Strikingly, most of the symptoms can already be seen when *MECP2* is deleted only in GABAergic cells (Chao et al., 2010), resulting in a defect of GABAergic synapses (Medrihan et al., 2008). This underlines the functional relevance of DNA methylation in GABAergic interneurons for neuropsychiatric diseases.

Changed DNA methylation signatures in GABAergic interneurons were further reported for schizophrenia patients, with genes like *REELIN* and *GAD1*, relevant for GABAergic neurotransmission as well as interneuron function and development, displaying elevated DNA methylation levels and reduced expression, being suggested to account for impaired interneuron function (Veldic et al., 2004; Ruzicka et al., 2007).

That DNMT1-dependent DNA methylation affects cortical interneuron functionality, was recently shown in mice. *Dnmt1* deletion in PV interneurons leads to increased inhibition, which however, seems not to be primarily caused by DNA methylation-dependent regulation of GABA- or synapse-related genes (Pensold et al., 2020). Instead, repressive DNMT1-dependent DNA methylation was identified to act on endocytosis-related gene expression in parvalbuminergic cortical interneurons. Functional validation experiments proposed that DNA methylation catalyzed by DNMT1 restricts clathrin-mediated endocytosis at pre-synapses, and through this synaptic vesicle recycling and GABAergic transmission (Pensold et al., 2020). Data obtained from human hippocampal biopsies of patients with temporal lobe epilepsy revealed a correlation between DNA methylation-dependent transcriptional regulation of endocytosis-related genes with the patients’ seizure rates (Pensold et al., 2020). This corroborates the connection between DNA methylation, endocytosis regulation and synaptic functionality.

DNA methylation-dependent regulation of endocytosis represents a novel mechanism for orchestrating synaptic transmission and might help us understand the pathophysiology of disorders characterized by abnormal synaptic transmission, as seen in different neuropsychiatric disorders (Ramocki and Zoghbi, 2008; Südhof, 2008). In support of this, it was recently reported that in addition to pyramidal neurons, PV cells in the DLPFC of schizophrenia patients display significant transcriptional changes of genes related to clathrin-mediated endocytosis signaling (Enwright et al., 2018). Another study analyzed genome-wide DNA methylation signatures in the frontal cortex of subjects diagnosed with schizophrenia and control subjects (Wockner et al., 2014). In cortical tissue of schizophrenia patients, significant DNA methylation changes were determined for genes related to endocytosis, including *CLTC*, *DNM1*, *DNM3*, *RAB7A*, *WIPF1*, *ZFYVE9*, *HGS*, *SPG20* and *RAC1* (Wockner et al., 2014). These genes were identified to be transcriptionally regulated by DNMT1-dependent DNA methylation in cortical PV interneurons in mice (Pensold et al., 2020). This proposes that DNA methylation-mediated alterations in endocytosis-related gene expression seem to be implicated in the pathophysiology of schizophrenia, which could consequently contribute to the impaired synaptic functionality in addition to the reported changes in GABA-related gene expression. However, the latter could alternatively represent a secondary consequence of dysregulated DNMT function to compensate for endocytosis-mediated alterations of synaptic transmission.

The observed changes in DNA methylation signatures in schizophrenia were hypothesized to be mediated by increased expression of *DNMT1*, as elevated *DNMT1* mRNA levels were found in post mortem schizophrenic brains (Veldic et al., 2004; Dong et al., 2015). However, a general increase in *DNMT1* expression does not explain site-specific effects. Alternatively, changes in DNMT1 targeting to specific gene loci could account as disease causing mechanisms. Site-specific DNA methylation by DNMT1 was proposed to be mediated by long non-coding RNAs (lncRNAs), preventing or promoting DNMT1 binding (Chalei et al., 2014). Indeed, there is accumulating evidence for the significance of lncRNAs impairment in several neuropsychiatric diseases including ASD, schizophrenia, intellectual disability, major depressive disorder and others (Hosseini et al., 2019).

Moreover, it should be kept in mind that DNMT1 was described to act non-canonically on gene expression by interactions with histone modifiers (Symmank and Zimmer, 2017; Symmank et al., 2018). DNMTs and DNA methylation interfere at different levels with histone modifications, which likewise modulate the specificity of transcriptional changes. Hence, a more global and combinatorial analysis of expression profiles and epigenetic signatures of post mortem brain material, favorably at single cell level, might help to get better insights into the role of DNMTs in mediating the patho-mechanisms of schizophrenia and other neuropsychiatric conditions at subcellular level.

## Discussion and Conclusion

For numerous neuropsychiatric diseases, abnormalities of the GABAergic system were identified. In fact, a dysfunction or dysgenesis of inhibitory interneurons in mouse models were observed to elicit pathological phenotypes, such as multimodal integration impairments. This coincides with inhibitory interneurons being pivotal for multisensory processing in the cerebral cortex. Next to genetic predispositions, environmental impact during development and resulting changes in the epigenetic landscape have more recently been considered as contributors to the diseases’ cause and course. Alterations in gene expression, relevant for cortical interneuron functionality, have been related to changes in DNA methylation signatures and DNMT1 expression levels in schizophrenia patients. Also, a mouse model of maternal adversity found that neonatal stress induced DNA methylation changes of genes related to synapse formation and function (Oh et al., 2013; Tordjman et al., 2014). Phenotypically, these mice showed neuropsychiatric symptoms, such as anxiety, elevated stress reactivity, and impairments of vocal communication, which could be affected by malfunctioning multimodal integration. Hence, a connection of epigenetic modulation and multisensory processing is highly likely and a noteworthy matter of future research.

DNMT1-dependent DNA methylation likewise regulates interneuron function in the healthy (mouse) brain, acting on synaptic transmission by modulating endocytosis, which might be affected in disease. Deciphering disease-relevant mechanisms requires more integrative analyses of patients’ brain samples, including the profiling of both, transcriptomic as well as epigenomic signatures. Therefore, studies analyzing changes in DNA methylation patterns need to be complemented by histone modification profiling and lncRNA expression and interaction analysis, to draw a conclusive picture of the hierarchy of epigenetic networks. Moreover, cell type-specific investigation of excitatory and inhibitory neurons of the different cortical layers within the distinct brain regions is elementary, to approach network changes and dynamics.

To relate the transcriptome to electrophysiological properties of cells, Patch-Sequencing techniques reach increasing popularity, which enables to correlate individual firing properties and eventually morphology with molecular features (Cadwell et al., 2016; Fuzik et al., 2016). The ongoing improvement of sequencing-based single cell approaches might render the comprehensive and parallel analysis of the epigenome, transcriptome, and electrophysiological features tangible. This will help to decipher which epigenetic mechanisms act as drivers or passengers in neuropsychiatric diseases.

## Author Contributions

Both authors were responsible for the article’s conceptual design, and wrote the manuscript, and agree to be accountable for the content of the work.

## Conflict of Interest

The authors declare that the research was conducted in the absence of any commercial or financial relationships that could be construed as a potential conflict of interest.
